# Grape Seed Proanthocyanidin Extract Alleviates AflatoxinB_1_-Induced Immunotoxicity and Oxidative Stress via Modulation of NF-κB and Nrf2 Signaling Pathways in Broilers

**DOI:** 10.3390/toxins11010023

**Published:** 2019-01-07

**Authors:** Shahid Ali Rajput, Lvhui Sun, Ni-Ya Zhang, Mahmoud Mohamed Khalil, Zhao Ling, Li Chong, Shuai Wang, Imran Rashid Rajput, Dost Muhammad Bloch, Farhan Anwar Khan, Aftab Shaukat, Desheng Qi

**Affiliations:** 1 Department of Animal Nutrition and Feed Science, College of Animal Science and Technology, Huazhong Agricultural University, Wuhan 430070, China; dr.shahidali@hotmail.com (S.A.R.); lvhuisun@mail.hzau.edu.cn (L.S.); zhangniya@mail.hzau.edu.cn (N.-Y.Z.); lingzhao@webmail.hzau.edu.cn (Z.L.); chrisli@webmail.hzau.edu.cn (L.C.); wangshuai@mail.hzau.edu.cn (S.W.); 2 Animal Production Department, Faculty of Agriculture, Benha University, Banha 13736, Egypt; mahmoud.khalil@fagr.bu.edu.eg; 3 Department of Biotechnology, Lasbela Univesity of Agriculture Water and Marine Science, Uthal, Balochistan 89250, Pakistan; drimranrajput@gmail.com (I.R.R.); vc@luawms.edu.pk (D.M.B.); 4 Department of Animal Health, Faculty of Animal Husbandry and Veterinary Sciences, University of Agriculture, Peshawar 25120, Pakistan; farhan82@aup.edu.pk; 5 College of veterinary medicine, Huazhong Agricultural University, Wuhan 430070, China; aftab.shaukat@webmail.hzau.edu.cn

**Keywords:** Aflatoxin B_1_, Grape Seed Proanthocyanidin Extract, Immunotoxicity, NF-κB, oxidative stress, Nrf2, Broilers

## Abstract

Aflatoxin B_1_ (AFB_1_) is a widely spread mycotoxin contaminates food and feed, causing severe oxidative stress damages and immunotoxicity. Grape seed proanthocyanidin (GSPE), a natural antioxidant with wide range of pharmacological and medicinal properties. The goal of the present study was to investigate the protective effects of GSPE against AFB_1_-induced immunotoxicity and oxidative stress via NF-κB and Nrf2 signaling pathways in broiler chickens. For the experiment, 240 one-day old Cobb chicks were allocated into four dietary treatment groups of six replicates (10 birds per replicate): 1. Basal diet (control); 2. Basal diet + AFB_1_ 1mg/kg contaminated corn (AFB_1_); 3. Basal diet + GSPE 250 mg/kg (GSPE); 4. Basal diet + AFB_1_ 1 mg/kg + GSPE 250 mg/kg (AFB_1_ + GSPE). The results showed that GSPE significantly decreased serum inflammatory cytokines TNF-α, IFN-γ, IL-1β, IL-10, and IL-6 induced by AFB_1_. Similarly, GSPE + AFB_1_ treated group revealed a significant decrease in mRNA expressions of pro-inflammatory cytokines (TNF-α, IFN-γ, IL-1β, and IL-6) in the splenic tissue compared to the AFB_1_ treatment group. In addition, western blotting results manifested that GSPE treatment normalized the phosphorylation of nuclear factor kappa B (p65) and the degradation of IκBα protein induced by AFB_1_. Furthermore, GSPE enhanced the antioxidant defense system through activating the nuclear factor-erythroid-2-related factor (Nrf2) signaling pathway. The mRNA and protein expression level of Nrf2 and its down streaming associated genes were noted up-regulated by the addition of GSPE, and down-regulated in the AFB_1_ group. Taken together, GSPE alleviates AFB_1_-induced immunotoxicity and oxidative damage by inhibiting the NF-κB and activating the Nrf2 signaling pathways in broiler chickens. Conclusively, our results suggest that GSPE could be considered as a potential natural agent for the prevention of AFB_1_-induced immunotoxicity and oxidative damage.

## 1. Introduction

Mycotoxins are hazardous to human and animals health worldwide because of their frequent occurrence in food and/or feedstuffs, and high probability to affect human health, and livestock and poultry productivity [1,2]. Aflatoxins are the widely studied mycotoxins, mainly produced by *Aspergillus flavus* and *Aspergillus parasiticus* [3]. The most common natural aflatoxins found in food and/or feed materials are B_1_, B_2_, G_1,_ and G_2_ type. Among the major aflatoxins, Aflatoxin B_1_ (AFB_1_) is reported as a widespreading and highly toxic type of aflatoxins [4,5,6]. AFB_1_ can affect several organs at a time, and classified as a Group-1 carcinogen to humans, with no safe dose [7]. Meanwhile, it has hepatotoxic, immunotoxic, carcinogenic, mutagenic, teratogenic, and other adverse health effects on humans and animals [8,9,10]. In poultry, AFB_1_ can severely affect the immune system, AFB_1_ immunosuppressive nature is the well-documented area of its toxicity [11]. Simultaneously, AFB_1_ can alter the size of the immune system organs, hence and severely altering the immunological functions in chickens [12]. The spleen is the largest lymphoid organ in the body, with a large number of T and B lymphocytes, and plays a vital role in the protective immune reactions [13]. Meanwhile, its functional ability in the generation, maturation, and storage of lymphocytes plays an important role in the humoral and cellular immune responses [14].

NF-κB is a transcription factor that has an essential role in cell proliferation, immunity inflammation, and oxidative stress [15,16,17]. Previously it was found that AFB_1_ combined with ochratoxin A (OTA) could exacerbate the immunotoxicity through the NF-κB signaling pathway [18]. However, antioxidant, anti-inflammatory and immunomodulatory properties of GSPE has been reported [19,20]. Therefore, it indicates that GSPE could play an important role in protecting the spleen immune injury from the damage caused by AFB_1_.

AFB_1_ increases the production of free radicals, lipid peroxidation, augments the oxidative damage, resulting in severe cell damage and/or cell death cycle to animals or humans. Oxidative stress has been reported to play a major role in the toxicity mechanism of AFB_1_ [21,22], whereas, the liver is considered the principal target organ for aflatoxins which is major metabolizing and detoxifying organ in the body [23,24]. Several studies have demonstrated that dysregulation of the Nrf2 signaling pathway is associated with AFB_1_-induced oxidative damage [25,26].

The Nrf2 signaling pathway has anti-oxidative effects by alleviating toxicant-induced oxidative stress damage [27]. It has been suggested that the anti-oxidative stress system can be activated through the Nrf2 signaling pathway, hence leads to excrete the toxic metabolites through the regulation of many intracellular antioxidant genes expression [28,29]. The Nrf2 is a primarily expressed gene within the metabolically active organs such as the liver. Therefore, the Nrf2 pathway is considered the most important therapeutic target for the prevention and treatment of oxidative stress-induced liver and its associated diseases [30,31,32].

Phytochemicals produced by plants through primary and secondary metabolism that have potential to activate the Nrf2 signaling pathway which may produce beneficial protecting effects against AFB_1_-induced liver damages [33,34,35]. Proanthocyanidins are one of the natural compounds that can found in fruits, seeds, vegetables, nuts, flowers, cereals and bark. Grape seed proanthocyanidin extract (GSPE) derived from grape seed, enriched with oligomeric proanthocyanidin, polymerized and polyphenolic flavonoids [36,37]. GSPE has potency as a powerful antioxidant for its ability to scavenge free oxygen radicals, with anti-cancer and anti-inflammatory effects [38]. GSPE has proven to have protective effect against zearalenone-induced hepatic injury and oxidative stress in the liver of Kunming mice [19]. Meanwhile, GSPE can protect the functions of major organs by improving the antioxidant system, and play a preventive role against carbon tetrachloride and ischemia/reperfusion [39,40]. Moreover, GSPE improves suppressed immune response induced by AFB_1_ in the spleen of mice [20]. In addition, GSPE demonstrated to have the ability to ameliorate lead-induced liver oxidative damage via the Nrf2/antioxidant response element (ARE) pathway [41].

In our previous study, we reported that GSPE had the ability to ameliorate oxidative damage induced by AFB_1_ in broiler chickens [42]. However, the precise molecular mechanism by which GSPE attenuates oxidative damage and immune injury caused by AFB_1_ in broiler chickens remained unclear. In the present study, we hypothesized that (1) GSPE will protect the spleen from AFB_1_-induced immune injury by suppressing the inflammatory response and inhibiting the NF-κB expression in broilers. (2) The protective effects of GSPE against AFB_1_-induced oxidative damage might be through regulating the Nrf2 signaling pathway in the liver of broiler chickens. Presumably, this is the first study to investigate the role of NF-κB and Nrf2 in the protective effects of GSPE against AFB_1_-induced immune injury and oxidative damage in broiler chickens.

## 2. Results

### 2.1. Serum Inflammatory Cytokines

**Figure 1:**
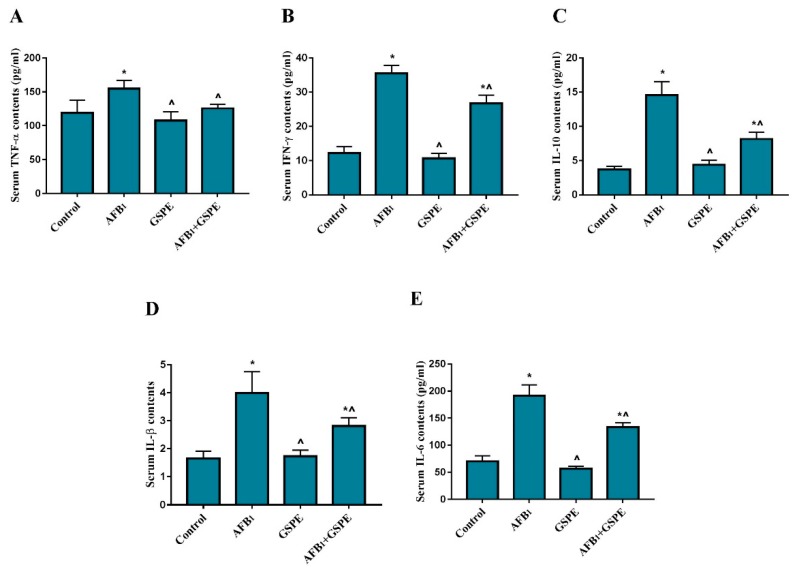
Protective role of grape seed proanthocyanidin extract (GSPE) treatment on Aflatoxin B_1_ (AFB_1_)-induced levels of the inflammatory cytokines in the serum of broilers. All data were expressed as mean ± SD (*n* = 6). Different symbols among groups indicate significant difference by least significant difference test (LSD) test (*p* < 0.05). * (*p* < 0.05) significant differences compared to the control group and ^ (*p* < 0.05) significant differences compared to the AFB_1_ group. (**A**) Tumor necrosis factor alpha (TNF-α); (**B**) interferon gamma (IFN-γ); (**C**) interleukin-1 beta (IL-1β); (**D**) interleukin 10 (IL-10); (**E**) interleukin 6 (IL-6).

The effect of GSPE supplementation on the levels of inflammatory cytokines in the serum of control and experimental groups of broilers are depicted in Figure 1. Broilers exposed to AFB_1_ showed (*p* < 0.05) increase in the serum TNF-α, IFN-γ, IL-1β, IL-10, and IL-6, as compared to the control group. However, AFB_1_ + GSPE co-treated group showed significant reduction in TNF-α, IFN-γ, IL-1β, IL-10, and IL-6, as compared to the AFB_1_ treated group. Besides this, single GSPE treatment group did not show any significant difference in the serum inflammatory cytokines as compared to the control group.

### 2.2. Pro-Inflammatory Cytokines Gene Expression

**Figure 2:**
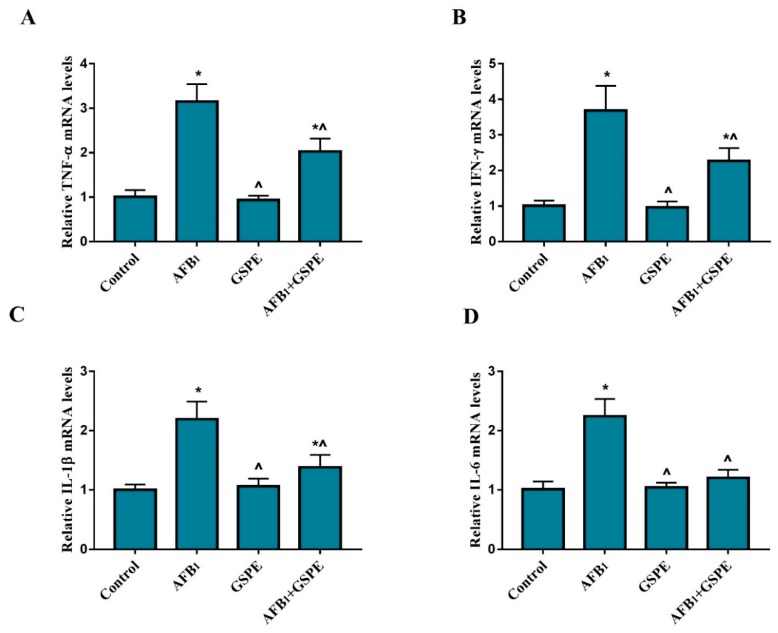
Protective role of GSPE treatment on AFB_1_-induced mRNA expression levels of the pro-inflammatory genes in the spleen of broilers. The mRNA expression of TNF-α, IFN-γ, IL-1β, and IL-6 were detected by quantitative real-time PCR. All data were expressed as mean ± SD (*n* = 6). Different symbols among groups indicate significant difference by LSD test (*p* < 0.05). * (*p* < 0.05) significant differences compared to the control group and ^ (*p* < 0.05) significant differences compared to the AFB_1_ group. (**A**) Tumor necrosis factor alpha (TNF-α); (**B**) interferon gamma (IFN-γ); (**C**) interleukin-1 beta (IL-1β); (**D**) interleukin 6 (IL-6).

To elucidate the protective role of GSPE on the AFB_1_-induced immune response, the gene expression of pro-inflammatory cytokines at mRNA levels were quantified by quantitative real-time PCR in the splenic tissue of broilers. As shown in Figure 2, the mRNA expressions of TNF-α, IFN-γ, IL-1β, and IL-6 were significantly up-regulated in the AFB_1_ group, as compared to the control group. However, the addition of GSPE to AFB_1_ contaminated diet (*p* < 0.05) revert the negative effect of AFB_1_ on the mRNA levels of TNF-α, IFN-γ, IL-1β, and IL-6, as compared to the AFB_1_ treated group. Accordingly, our results suggest that pro-inflammatory cytokines gene expression inhibited by GSPE and thereby inflammatory response alleviated in the spleen induced by AFB_1_ in broilers.

### 2.3. Effects of GSPE and AFB_1_ on the Degradation of IκBα and the Phosphorylation of NF-κB (p65)

**Figure 3:**
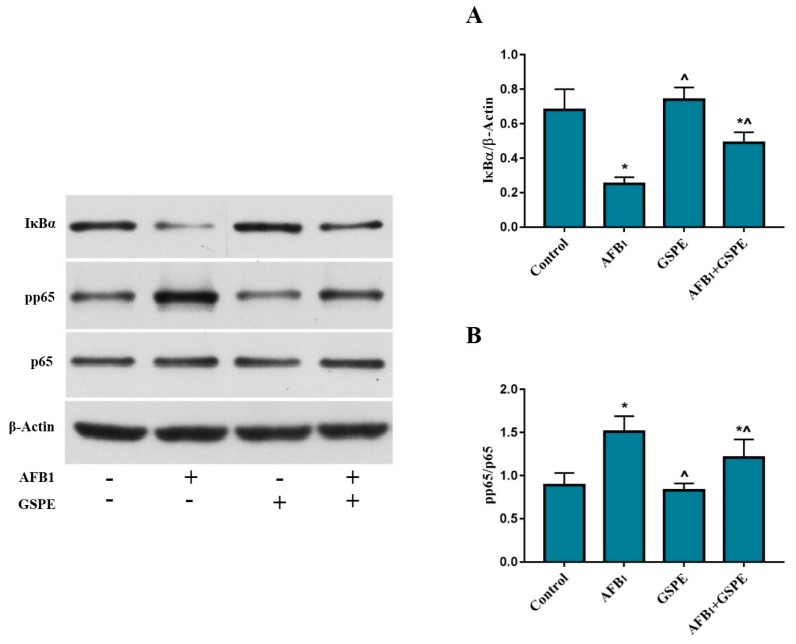
Protective role of GSPE treatment on the AFB_1_-induced degradation IκBα (**A**) and phosphorylation of NF-κB (p65) (**B**). The protein expression of β-actin, p65, pp65, and IκBα protein were detected by western blotting in the spleen of broilers. All data were expressed as mean ± SD. Different symbols among groups indicate significant difference by LSD test (*p* < 0.05). * (*p* < 0.05) significant differences compared to the control group and ^ (*p* < 0.05) significant differences compared to the AFB_1_ group.

The NF-κB signaling pathway is considered a key factor to monitor the genes responsible for the immune and inflammatory responses. To confirm our hypothesis that NF-κB signaling mechanism involves in immunotoxicity, that is induced by AFB_1_ in the spleen of broilers. In the current study, the western blotting approach was used to determine the AFB_1_ toxicity impact and inhibition by GSPE. We found that AFB_1_ caused a (*p* < 0.05) degradation in the IκBα protein as compared to the control group. However, supplementation of GSPE into AFB_1_ contaminated diet showed prominent (*p* < 0.05) inhibition of AFB_1_-induced IκBα protein degradation. Figure 3A. Furthermore, AFB_1_ significantly elevated the phosphorylation of NF-κB (p65), while the addition of GSPE into AFB_1_ contaminated diet significantly reduced the phosphorylation of NF-κB (p65) Figure 3B.

### 2.4. Nrf2 and Its Downstream Genes (HO-1, GPx1, NQO1, and GCLC) mRNA Expression

**Figure 4:**
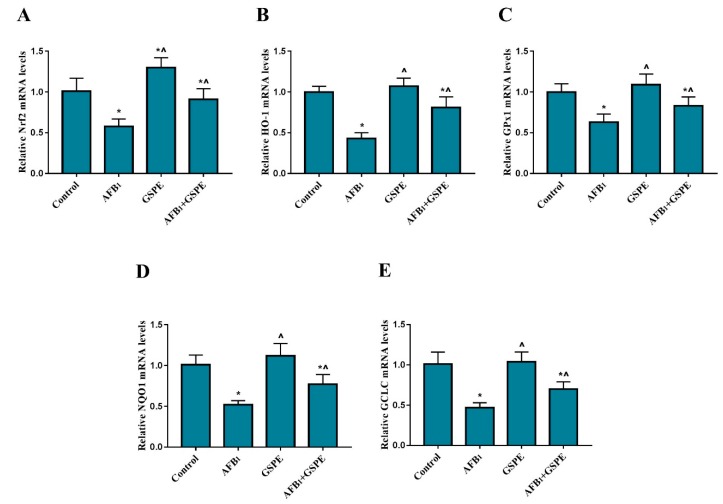
Protective role of GSPE treatment on AFB_1_-induced mRNA expression levels of the Nrf2 signaling pathway in the liver of broilers. The mRNA expression of Nrf2, HO-1, GPx1, NQO1, and GCLC were detected by quantitative real-time PCR. All data were expressed as mean ± SD (*n* = 6). Different symbols among groups indicate significant difference by LSD test (*p* < 0.05). * (*p* < 0.05) significant differences compared to the control group and ^ (*p* < 0.05) significant differences compared to the AFB_1_ group. (**A**) Nuclear erythroid-2-related factor (Nrf2); (**B**) heme oxygenase-1 (HO-1); (**C**) glutathione peroxidase (GPx1); (**D**) quinone oxidoreductase 1 (NQO1); (**E**) glutamate-cysteine ligase catalytic subunit (GCLC).

We assessed the protective effects of GSPE against AFB_1_-induced oxidative damage through regulating the Nrf2 signaling pathway in the liver of broiler chickens. The mRNA levels of HO-1, GPx1, NQO1, GCLC, and Nrf2 were examined by quantitative real-time PCR. The results of AFB_1_ treatment group showed (Figure 4) a significant decrease in the mRNA level of Nrf2 gene, as compared to the control group. The supplementation of GSPE to dietary treatments showed (*p* < 0.05) increase in the gene expression of Nrf2 in the liver of broilers by 22% and 56% when compared with the control and AFB_1_ group, respectively (Figure 4A). Moreover, compared to the control group, the mRNA expression level of HO-1, GPx1, NQO1, and GCLC genes in the AFB_1_ group were (*p* < 0.05) down-regulated. In contrast, the addition of GSPE to the AFB_1_ contaminated diet showed significant improvement in the mRNA expression levels of HO-1, GPx1, NQO1, and GCLC decreased by AFB_1_ Figure 4B–E.

### 2.5. Nrf2 and Its Downstream Genes (HO-1, GPx1, NQO1, and GCLC) Protein Expression

**Figure 5:**
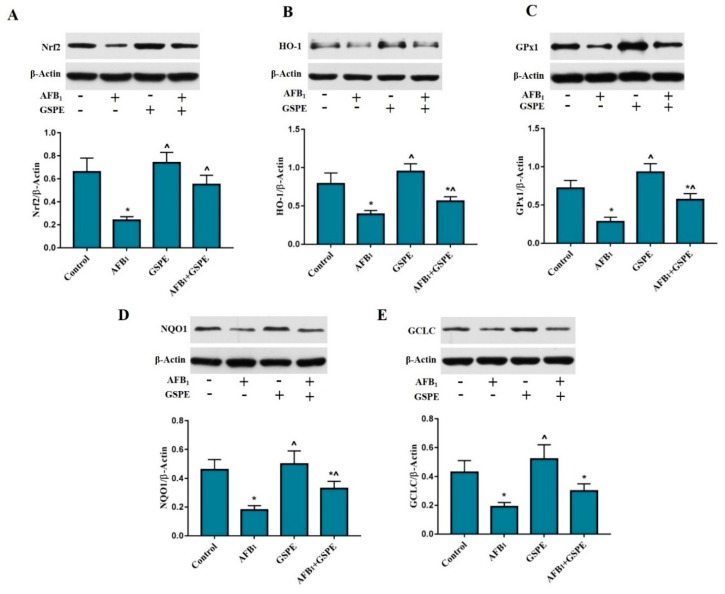
Protective role of GSPE treatment on AFB_1_-induced protein expression levels of the Nrf2 signaling pathway in the liver of broilers. The protein expression of β-actin, Nrf2, HO-1, GPx1, NQO1, and GCLC were detected by western blotting. All data were expressed as mean ± SD. Different symbols among groups indicate significant difference by LSD test (*p* < 0.05). * (*p* < 0.05) significant differences compared to the control group and ^ (*p* < 0.05) significant differences compared to the AFB_1_ group. (**A**) Nuclear erythroid-2-related factor (Nrf2); (**B**) heme oxygenase-1 (HO-1); (**C**) glutathione peroxidase 1 (GPx1); (**D**) quinone oxidoreductase 1 (NQO1); (**E**) glutamate-cysteine ligase catalytic subunit (GCLC).

To understand whether the protective effects of GSPE against AFB_1_-induced oxidative damage is associated with the Nrf2 gene activation, we measured the protein level of Nrf2 and its target proteins, HO-1, GPx1 NQO1, and GCLC in broiler liver by western blotting (Figure 5). Exposure to AFB_1_ resulted in the down-regulation of the Nrf2 protein expression, and this effect was alleviated by the addition of GSPE into diets contaminated with AFB_1_, with a significant up-regulation of the Nrf2 protein expression when compared to the AFB_1_ treated group (Figure 5A). Furthermore, we noted that protein expression of HO-1, GPx1, NQO1, and GCLC showed a (*p* < 0.05) reduced in the AFB_1_ fed group, as compared to the control group. While the supplementation of dietary GSPE into AFB_1_ contaminated diet significantly ameliorated HO-1, GPx1, and NQO1 protein expressions when compared to the AFB_1_ group Figure 5B–D. However, the numerical difference was found in GCLC protein expression, as compared to the AFB_1_ group.

## 3. Discussion

The immunosuppressive nature of AFB_1_ is a well-supported area of its severe toxicity, and specifically, in poultry the immune system of birds is severely affected by AFB_1_ [11]. In the present study, a significant increase in inflammatory cytokines TNF-α, IFN-γ IL-1β, IL-10, and IL-6 in AFB_1_ group was observed in the serum of broilers, as compared to the control group. These results are in consistency with previous reports which indicated that AFB_1_ could lead to inflammation and alter the immune response [20,43]. However, the level of TNF-α, IFN-γ IL-1β, IL-10, and IL-6 remained lower in the normal physiological state, comparing with extrinsic effects. Meanwhile, cytokines are secreted and released by affected cells and released into the blood, which increases the levels of cytokines in the serum, indicating inflammation of tissue. Previously it was reported that GSPE could significantly improve the serum inflammatory cytokines induced by AFB_1_ in the mice [20]. The findings were similar to our results, which showed that the addition of GSPE to diets contaminated with AFB_1_ significantly ameliorated TNF-α, IFN-γ IL-1β, IL-10, and IL-6 induced by AFB_1_ in the serum of broilers.

In avian species, the spleen is the peripheral lymphoid organ, which plays a major protective role against inflammation and acquired immune response. The pro-inflammatory cytokines such as TNF-α, IFN-γ IL-1β, and IL-6 could trigger immune responses, as well as interrelate cells in order to eliminate the toxic effects and actively release other inflammation mediators [13]. Therefore, we determined the mRNA expression of the pro-inflammatory cytokines within the spleen, which can reflect the immune status of the broiler chickens. The current study showed that mRNA expression of TNF-α, IFN-γ IL-1β, and IL-6 were increased in the spleen of broilers exposed to AFB_1_. Moreover, our results indicated that the inclusion of AFB_1_ in the diet leads to an inflammation response in the spleen, similar effects had been observed in previous studies [20,43,44]. However, GSPE inhibited the mRNA expression of pro-inflammatory cytokines IL-1β, IL-6, TNF-α, and IFN-γ, of spleens induced by AFB_1_ in broilers. These results are consistent with those of [45,46,47] who have demonstrated that GSPE has anti-inflammatory effects in various animal models. Our findings indicated that GSPE has anti-inflammatory properties via modulating the secretion of pro-inflammatory cytokines.

Nuclear factor- κB (NF-κB) is a transcriptional factor that regulates immune and inflammatory responses in various condition [48,49]. In the current study, we found that the immunotoxicity induced by AFB_1_ promoted the phosphorylation of the NF-κB and the degradation of the IκBα protein. However, AFB_1_ toxicity was significantly ameliorated by the GSPE treatment. NF-κB is isolated from the cytoplasm through direct interaction with one of the inhibitor proteins of the IκB family such as IκBα. The phosphorylation and degradation of the IκBα proteins showed to be essential for the activation of the NF-κB, hence leads to a rapid translocation of NF-κB from the cytoplasm to the nucleus [50]. Our results are in accordance with the previous study [18], revealing that the presence of AFB_1_ in combination with OTA could aggravate immunotoxicity through the NF-κB signaling pathway. Furthermore, the addition of GSPE into AFB_1_ contaminated diet blocked the phosphorylation of NF-κB and the degradation of IκBα protein, which was the primary protein to activate NF-κB. The present study results indicated that the activation of NF-κB might be the reason for the spleen immune injury and inflammatory response in broilers. Our current study findings are suggesting that the mitigation of NF-κB activation might be responsible for the protective effects of GSPE against immunotoxicity of broilers induced by AFB_1_.

The Nrf2 signaling pathway has anti-oxidative effects on alleviating toxicant-induced oxidative stress and hepatotoxicity [27]. Activation of the Nrf2 signaling pathway enables to protect the cells from the oxidative damage. The antioxidative stress system can be activated by the Nrf2 signaling pathway, in parallel with regulating the expression of many intracellular antioxidant genes leads to excrete toxins [28,29]. Nrf2 and its target genes such as HO-1, GSH-Px, NQO1, and GCLC are critical components of the endogenous redox system. These proteins have been known to have the cytoprotective resistant effect to oxidative stress [51,52,53,54]. In our previous work, we found that GSPE could ameliorate AFB_1_-induced hepatotoxicity and oxidative damage in the liver of broiler chickens [42]. However, the precise mechanism by which GSPE attenuates oxidative damage caused by AFB_1_ in broiler chickens remained unclarified. Therefore, in the current study, we investigated the protective effects of GSPE on AFB_1_-induced oxidative damage through regulating the Nrf2 signaling pathway in the liver of broiler chickens. In the present study, the addition of GSPE to AFB_1_ contaminated diet attenuated the AFB_1_-induced decrease in Nrf2 in both mRNA and protein expression in the liver of broilers. Moreover, the mRNA and protein expressions of HO-1, GPx1, NQO1, and GCLC were up-regulated by the addition of GSPE to AFB_1_ diet. Our results indicated that GSPE could activate the Nrf2, resulting in enhancing the expression of HO-1, GPx1, NQO1, and GCLC, leading to improve the oxidative stress resistance, hence maintain the redox balance and enhance the resistance ability to oxidative stress induced by AFB_1_ in the liver of broilers. The present study results are continuity of previous studies that showed GSPE could ameliorate toxicant-induced oxidative damage via activating the Nrf2 signaling pathway. Long et al. [19], reported that GSPE attenuated the zearalenone-induced oxidative damage in the Kunming mice and the mechanism was related to the activation of the Nrf2 signaling pathway. Moreover, GSPE can induce Nrf2 expression and ARE-mediated transcription, thereby reducing oxidative stress. For instance, GSPE inhibited lead-induced liver oxidative damage and elevated antioxidant capacity via the activation of the Nrf2/ARE signaling pathway [41]. Furthermore, oligomeric proanthocyanidins markedly enhanced the nuclear translocation of Nrf2, promoted the expression of HO-1, NQO1, and thioredoxin reductase 1, and suppressed H_2_O_2_-induced oxidative damage in A549 cells [55]. The present study revealed that the protective effect of GSPE against AFB_1_ induced oxidative damage might be triggering the Nrf2 signaling pathway.

## 4. Conclusions

In summary, our findings indicated that the exposure to AFB_1_ could exacerbate inflammatory response, immunotoxicity and oxidative damage in broilers. Conversely, GSPE protects the immunotoxicity and oxidative damage AFB_1_-induced through the modulation of the NF-κB and Nrf2 signaling pathways. Taken together, our data demonstrated that NF-κB and Nrf2 responses were modulated by GSPE, which indicated the potential application of GSPE against immunotoxicity and oxidative stress in broiler chickens. Moreover, our findings propose a potential explanation for the mechanism of the antioxidant and immunomodulatory activities of GSPE and could be considered as a potential natural agent for the prevention of AFB_1_-induced immunotoxicity and oxidative damage in humans and animals.

## 5. Material and Methods

### 5.1. Fungal Isolation

In the present study *Aspergillus flavus* (NRRL-3357) strain was used for AFB_1_ production [56]. The strain was maintained as a glycerol stock preparation at −80 °C. It was grown on Petri dishes containing potato dextrose agar (E. Merck, Darmstadt, Germany) medium at 30 °C for seven days.

### 5.2. Aflatoxin B_1_ Production

Aflatoxin B_1_ was produced in corn by inoculating the *Aspergillus flavus* (NRRL-3357) according to the technique proposed by [57]. Briefly, twenty grams of ground maize were placed in Erlenmeyer flasks, then autoclaved at 121 °C for 20 min to eliminate their natural micro-flora. Each flask was inoculated by *Aspergillus flavus* containing 1 × 10^6^ spores/grams. Mature spores were harvested with sterile 0.85% physiological saline. The moisture content was adjusted to the desired level (20%) by adding the necessary calculated volume of sterilized distilled water. The flasks were sealed with plastic film, allowing gaseous and vapor exchange, and then were mixed thoroughly by vigorous shaking to obtain a homogeneous distribution of the inoculum. The flasks were incubated under stationary conditions in a constant temperature and humidity incubator at 30 ± 1 °C and relative humidity (85%) for 15 days. Each flask was shaken once a day. The inoculated corn was incubated for 15 days to obtain the approximate AFB_1_ content of 64 mg/kg. The AFB_1_ contaminated corn was stored at 4 °C prior to treatment.

### 5.3. Aflatoxin B_1_ Analysis

The concentration of aflatoxin B_1_ was detected by High-Performance Liquid Chromatography (Waldbronn, Germany) equipped with a fluorescence detector and chromatographic separation was achieved using a C_18_ column (250 × 4.6 mm, 5 µm, Agilent, Santa Clara, CA, USA) following the methodology proposed by [57].

### 5.4. Compliance with Ethical Standards

All the experiments procedures were approved on 7 August 2017 (approval No. HZAUCH-2017-007) and conducted under the guidelines provided by the Institutional Animal Care and Ethics Committee of Huazhong Agricultural University, Wuhan, China.

### 5.5. Bird, Diets, and Management

GSPE was purchased from Zelang Medical Technology Company (Nanjing, China; purity *≥* 98%). For the present experiment, 240 one-day old Cobb broilers were obtained from a commercial hatchery (Jingzhou Kang Poultry Co., Ltd., Jingzhou, China). After three days acclimation, birds with similar body weight were randomly divided into four groups with six replicates of ten birds each (*n* = 60 per group). Groups were allocated based on the following four dietary treatments; 1. Basal diet without addition of GSPE and AFB_1_ (Control), 2. Basal diet supplemented with AFB_1_ 1 mg/kg (AFB_1_), 3. Basal diet supplemented with GSPE 250 mg/kg (GSPE), 4. Basal diet supplemented with AFB_1_ 1 mg/kg + GSPE 250 mg/kg (AFB_1_ + GSPE). The doses of AFB_1_ and GSPE were chosen considering our previous study [42]. Birds were fed ad libitum and provided fresh drinking water during the whole experimental period (28 days). The experiment was conducted under standard temperature and hygienic conditions. The composition of the basal diet has been presented in Table 1.

### 5.6. Collection of Samples

At the 28th day of age, one bird close to the average weight was selected from each replicate. After chickens fasted for 12 h, blood samples were collected in tubes by puncture of the wing vein. The blood samples were centrifuged (Eppendorf centrifuge 5804R, Hamburg, Germany) at 3000× *g* at 4 °C for 10 min, and the serum was separated and stored at −20 °C for cytokines analysis. After taking blood samples, birds were euthanized by cervical dislocation, and spleen and liver were collected. The spleen and liver were snap frozen in liquid nitrogen and later stored at −80 °C for further analysis.

### 5.7. Determination of Serum Cytokines

The immune response in the serum was determined by measuring the levels of TNF-α (CSB-E11231Ch), IFN-γ (CSB-E08550Ch), IL-1β, IL-10 (CSB-E12835Ch), and IL-6 (CSB-E08549Ch). These cytokines were analyzed using the Enzyme Linked-Immunosorbent Assay (ELISA) method through the specific assay kits. The details of all the determination procedures followed by manufactures protocols for the commercial kits supplied by (Cusabio Biotech Co. Ltd., Wuhan, China). The levels of cytokines were expressed as picogram per milliliter (pg/mL).

### 5.8. Total RNA Extraction and Quantitative Real-Time PCR

Total mRNA was extracted from spleen and liver tissues with Trizol^®^ (Invitrogen, Carlsbad, CA, USA) according to the manufacturer’s instructions. The purity and concentration of RNA samples were estimated by nucleic acid concentration analyzer NanoDrop 2000 (Thermo Fisher, Waltham, MA, USA) based on the ratio of the absorbance at 260 and 280 nm. The cDNA was synthesized from 1 µg of total RNA by reverse transcription in a 20 µL reaction using a PrimeScript^TM^ RT reagent Kit (Takara DRR037A, Dalian, China) following the manufacturer’s protocol. The expression levels of pertaining genes (β-actin, TNF-α, IFN-γ, IL-1β, IL-10, IL-6, Nrf2, HO-1, GPx1, NQO1, and GCLC) were analyzed by quantitative real-time PCR (CFX384, Bio-Rad, Hercules, CA, USA) using the SYBER^®^ Green PCR Master Mix (Applied Biosynthesis, Waltham, MA, USA), following the method according to our previous study [58] and the manufacturer’s instructions. The primer sequences used for each gene are presented in Table 2. The 2^-∆∆Ct^ method was used for quantification with the β-actin as a reference gene, and the relative abundance was normalized to the control (as 1) [59,60]. The results were expressed as relative mRNA levels.

### 5.9. Western Blot Analysis

Protein expressions of Nrf2, HO-1, GPx1, NQO1 and GCLC in the liver and NF-κB, p-NF-κB, and IκBα in the spleen of broilers were determined by western blot according to our previous study [58]. The following antibodies used for the present study were purchased from indicated sources: Anti-NF-κB, Anti-p-NF-κB, Anti-Nrf2, Anti-HO-1, anti-GPx1, (Abcam, Cambridge, MA, USA), Anti-NQO1, GCLC (Abclonal Technology, Woburn, MA, USA), Anti-β-Actin, Anti-IκBα (Cell Signaling Technology, Boston, MA, USA). The HRP-labeled goat anti-rabbit IgG (Servicebio Technology, Wuhan, China) was used as the secondary antibody. Samples were analyzed in triplicate; a representative blot is shown in respective figures. The proteins bands were detected via chemiluminescence WesternBright^TM^ ECL substrate kit (Advansta, Menlo Park, CA, USA), then visualized and quantified by FluroChem FC2 Imaging System.

### 5.10. Statistical Analysis

The experimental data were analyzed by one-way ANOVA using IBM SPSS Statistic 22 (IBM Corporation, Armonk, New York, NY, USA). Differences were considered to be significant at *p* < 0.05, the least significant difference test (LSD) was used to separate the significant differences between means. Results were presented as mean ± SD.

## Figures and Tables

**Table 1:** Basal diet formulation and nutritional value.

Ingredients	Percentage %
Corn	58.3
Soybean meal	30.2
Fish meal	5.6
Soybean oil	2.3
Dicalcium phosphate	1.2
Lime stone	1.00
Salt	0.2
Methionine	0.2
Premix ^1^	1.00
Total	100.00
**Calculated chemical composition**	
Crude protein	21.87
Metabolisable energy (MJ/kg)	13.45
Lysine	1.14
Methionine	0.40
Methionine + Cystine	0.94
Calcium	0.95
Available phosphorus	0.49

^1^ The premix contained (per kg of diet): Fe, 60 mg; Cu, 7.5 mg; Zn, 65 mg; Mn, 110 mg; I, 1.1 mg; Se, 0.4 mg; Biotin, 0.04 mg; choline chloride, 400 mg; vitamin A (from retinyl acetate), 4500 IU; vitamin D3 (from cholecalciferol), 1000 IU; vitamin K (menadione sodium bisulphate), 1.3 mg; vitamin B1, 2.2 mg; vitamin B2, 10 mg; vitamin B3, 10 mg; vitamin B5, 50 mg; vitamin B6, 4 mg; vitamin B11, 1 mg; vitamin B12, 0.013 mg.

**Table 2:** Primer used for quantitative real-time PCR.

Target Gene	Primer	Primer Sequence (5′→3′)	Accession No.
β-Actin	Forward Reverse	CCCGCAAATGCTCTAAACC CCAATCCTGTCTTGTTTTATGC	L08165
IL-1β	Forward Reverse	GGTCAACATCGCCACCTACA CATACGAGATGCAAACCAGCAA	NM_204524.1
IL-6	Forward Reverse	GGTGATAAATCCCGATGAAGT TCTCCATAAACGAAGTAAAGTCTC	NM_204628
IFNγ	Forward Reverse	TGAGCCAGATTGTTTCGATG TCCTTTTGAAACTCGGAGGA	NM_205149
TNFα	Forward Reverse	TGTGTATGTGCAGCAACCCGTAGT GGCATTGCAATTTGGACAGAAGT	AY765397.1
Nrf2	Forward Reverse	GATGTCACCCTGCCCTTAG CTGCCACCATGTTATTCC	NM_205117
HO-1	Forward Reverse	GGTCCCGAATGAATGCCCTTG ACCGTTCTCCTGGCTCTTGG	HM237181.1
GPx1	Forward Reverse	GACCAACCCGCAGTACATCA GAGGTGCGGGCTTTCCTTTA	NM_001277853.1
NQO1	Forward Reverse	CAGTGGCATGCACCCAGGGAA GCATGCCCCTTTTAGCCTTGGCA	NM_001277619.1
GCLC	Forward Reverse	AGTGCTGAGTGGCGAAGAAGT GCAGCCTCTTGCCTCCTCTT	XM_419910.5
